# Supplementary material for: The Hindu Kush slab break-off as revealed by deep structure and crustal deformation
Source: Nat Commun. 2021 Mar 16;12:1685. doi: 10.1038/s41467-021-21760-w (PMC7966371; doi:10.1038/s41467-021-21760-w)
Supplement: Supplementary file 1 — Supplementary Information [file 41467_2021_21760_MOESM1_ESM.pdf]

Supplementary Information for
The Hindu Kush slab break-off as revealed by deep structure and crustal deformation

Sofia-Katerina Kufner (1/2/*†), Najibullah Kakar (3/4), Maximiliano Bezada (2), Wasja Bloch (1), Sabrina Metzger (1), Xiaohui Yuan (1), James Mechie (1), Lothar Ratschbacher (5), Shokhruhk Murodkulov (6), Zhiguo Deng (1), and Bernd Schurr (1)

(1) GFZ German Research Centre for Geosciences, Potsdam, Germany
(2) University of Minnesota, Minneapolis, USA
(3) Norwegian Afghanistan Committee, Kabul, Afghanistan
(4) University of Potsdam, Potsdam, Germany
(5) TU Bergakademie Freiberg, Freiberg, Germany
(6) Tajik Academy of Sciences, Dushanbe, Tajikistan

*Corresponding author: Sofia-Katerina Kufner, mail: sofner@bas.ac.uk, ORCID: 0000-0002-9687-5455

† now at British Antarctic Survey, Cambridge, United Kingdom

Content

- Supplementary Note 1. Joint inversion comparison to previous tomography studies

- Supplementary Figure 1 Inversion grid geometry.
- Supplementary Figure 2 Comparison of tomography models.
- Supplementary Figure 3 Checkerboard test.
- Supplementary Figure 4 Comparison of crustal event catalogues.
- Supplementary Figure 5 Stress inversion in sub-regions.
- Supplementary Figure 6 Input data for tomography separated by network deployment time.
- Supplementary Figure 7 Input picks for inversion and output station terms.
- Supplementary Figure 8 Trade-off curves between data variance and model variance dependent on different smoothing and damping values used in the inversion.

- Supplementary Table 1 Detailed explanation of input data subsets.

- Supplementary References

Supplementary Note 1. Joint inversion comparison to previous tomography studies

The comparison to either local or teleseismic tomography that had been calculated based on pre-2017 datasets is shown in Supplementary Fig. 2. Comparing the joint tomography presented in this study (Supplementary Fig. 2a) to previous local tomography studies (Supplementary Figs. 2b/c [1,2]) calculated with the widely used simulps code [3] shows generally similar absolute P-wave velocities (vP) in crustal regions resolved in all studies (e.g. western Pamir, eastern Afghan-Tajik depression). The joint model features slower absolute vP in the mantle compared to the local tomography models (e.g. 80 and 120 km depth). This effect might arise as the local inversions are based on faster reference velocities at these depths and less crossing local rays exist in the mantle (compared to crustal depths). However, these deviations of the joint tomography from the published studies are in the same range than second order variations in the two published models, highlighting the general resolution limits of local tomography.

Supplementary Figures 2d-f juxtapose the teleseismic only and joint model derived herein and a published teleseismic model [4] along profile 2d. Due to similar station coverage, both studies are equally well resolved along this profile. Despite different inversion codes (finite-frequency kernels (see “Methods”) vs. teleLOTOS [5]), grid geometries and input datasets, the two teleseismic models are very similar in the upper mantle.

In general, the velocity anomalies resolved in this study appear smoother compared to those resolved in other studies. This effect may arise as the grid geometry and smoothing and damping parameters in the joint tomography were tuned based on the combined local and teleseismic data. However, compared to previous studies, only the joint inversion approach is capable to recover both, crustal and mantle anomalies.

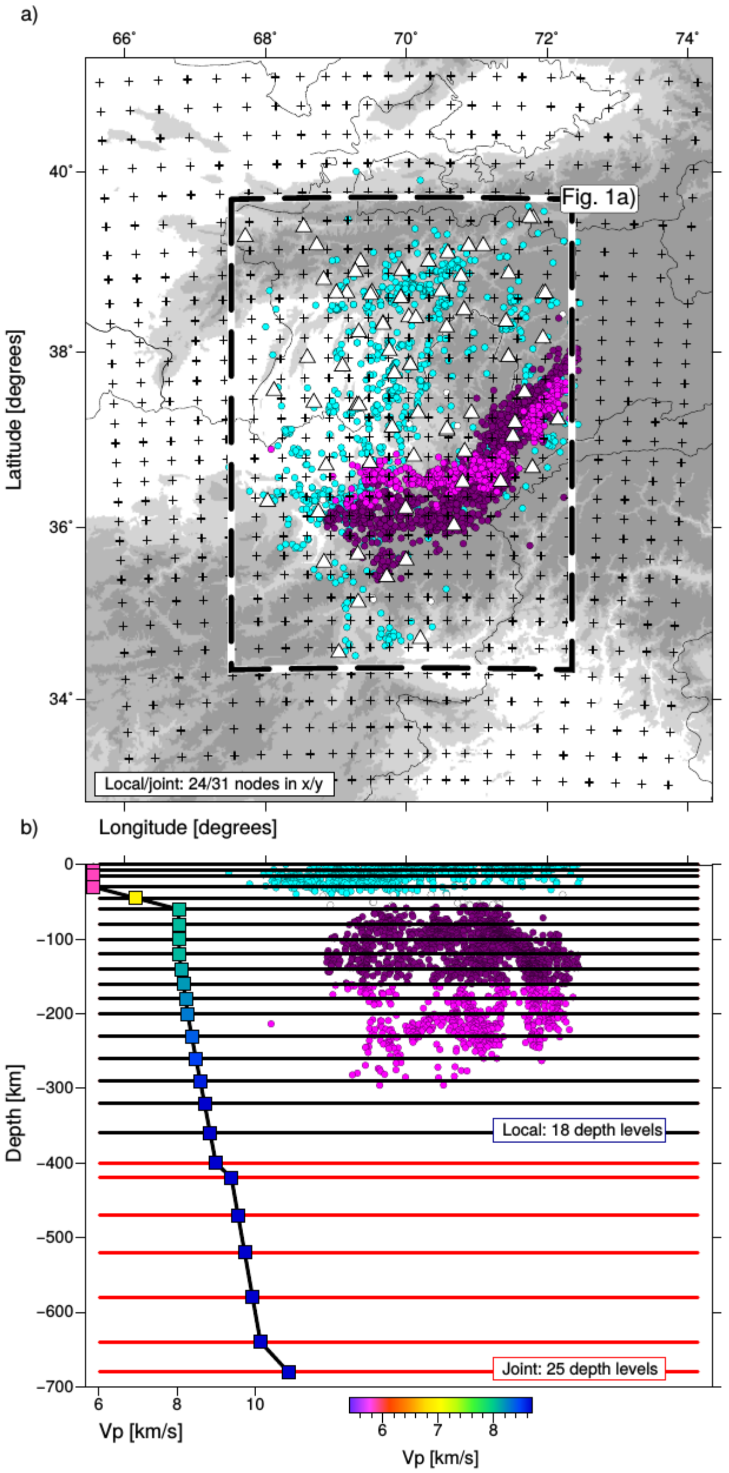

Supplementary Fig. 1 Inversion grid geometry. a) Map view; black crosses mark the grid used for the inversions. Stations (white triangles), local earthquakes (colour-coded circles) and topography as in Fig. 1a. Political boundaries in dark grey. b) Depth layers of inversion grid nodes and initial velocity model. Depth layers implemented in the teleseismic and joint inversion only are highlighted in red. Colour-coding of earthquakes (circles) as in Fig. 1a.

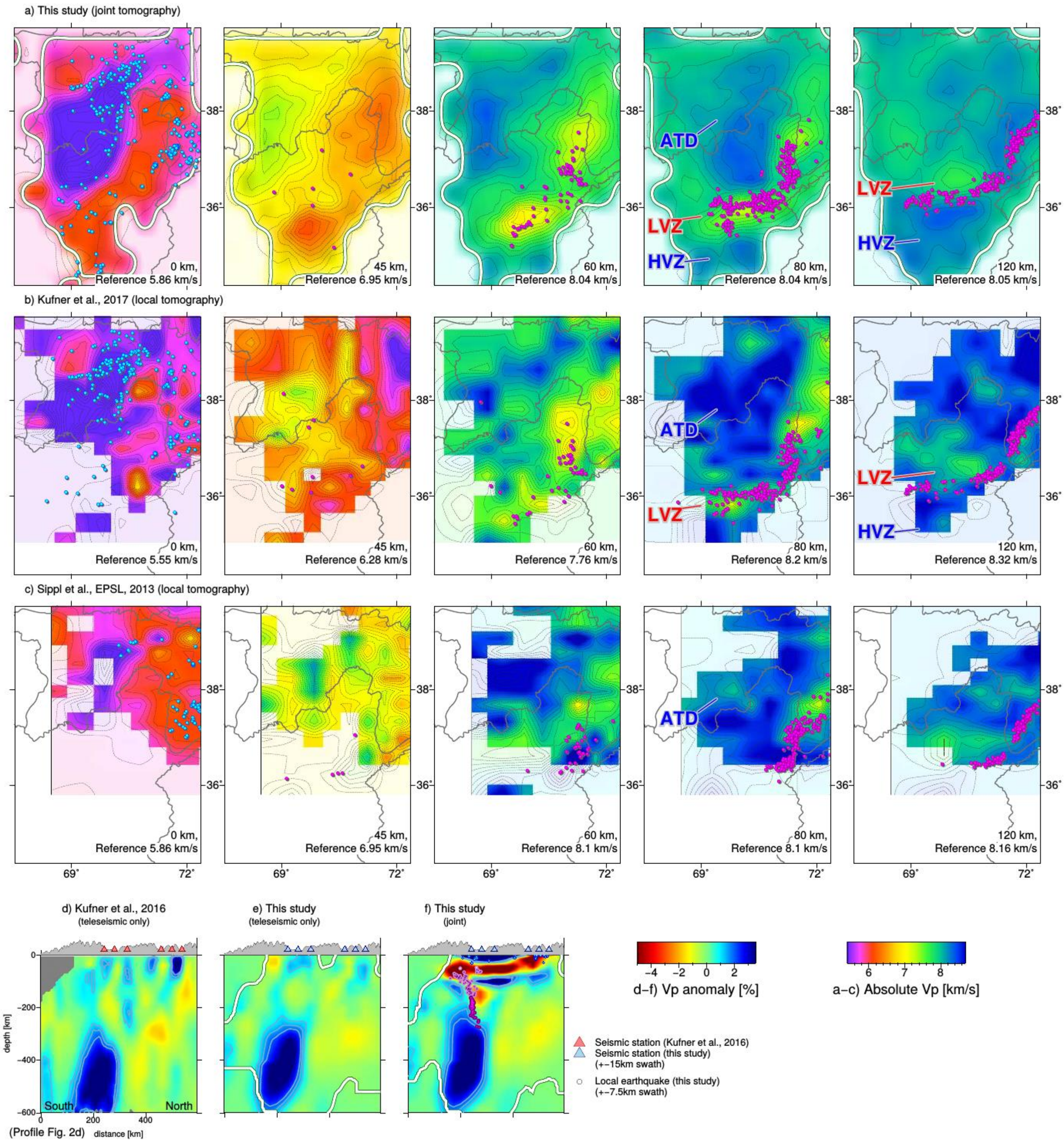

Supplementary Fig. 2 Comparison of tomography models. a) Absolute velocities in depth sections obtained in this study from the combined (joint) inversion of local and teleseismic data. White outline represents resolution limit as in Fig. 2. Contour interval (black) is 0.2 km/s. Local earthquakes projected onto depth maps as in Figs. 2f-k, but only events used in the inversion are shown. LVZ/HVZ/ATD refer to the anomalies discussed in this article. b) Local tomography model from ref. [2], derived using the simulps inversion code [3]. This inversion includes only a few stations and crustal earthquakes in Afghanistan. Only well-resolved model domains are colour-coded. Contouring as in a). c) Local tomography model from ref. [1] derived using simulps. This study focused on the Pamir and the eastern Afghan-Tajik depression. As in b) only well-resolved model domains are colour-coded. Contouring as in a/b). The reference velocity listed for each depth map refers to the initial velocity of the 1D velocity model used in the inversion. d) Teleseismic tomography from ref. [4] along the profile of Fig. 2d. The inversion was conducted using TeleLOTOS [5]. e/f) Teleseismic and joint model of this study along the same profile for comparison. All plotted features as in Figs. 2a-e.

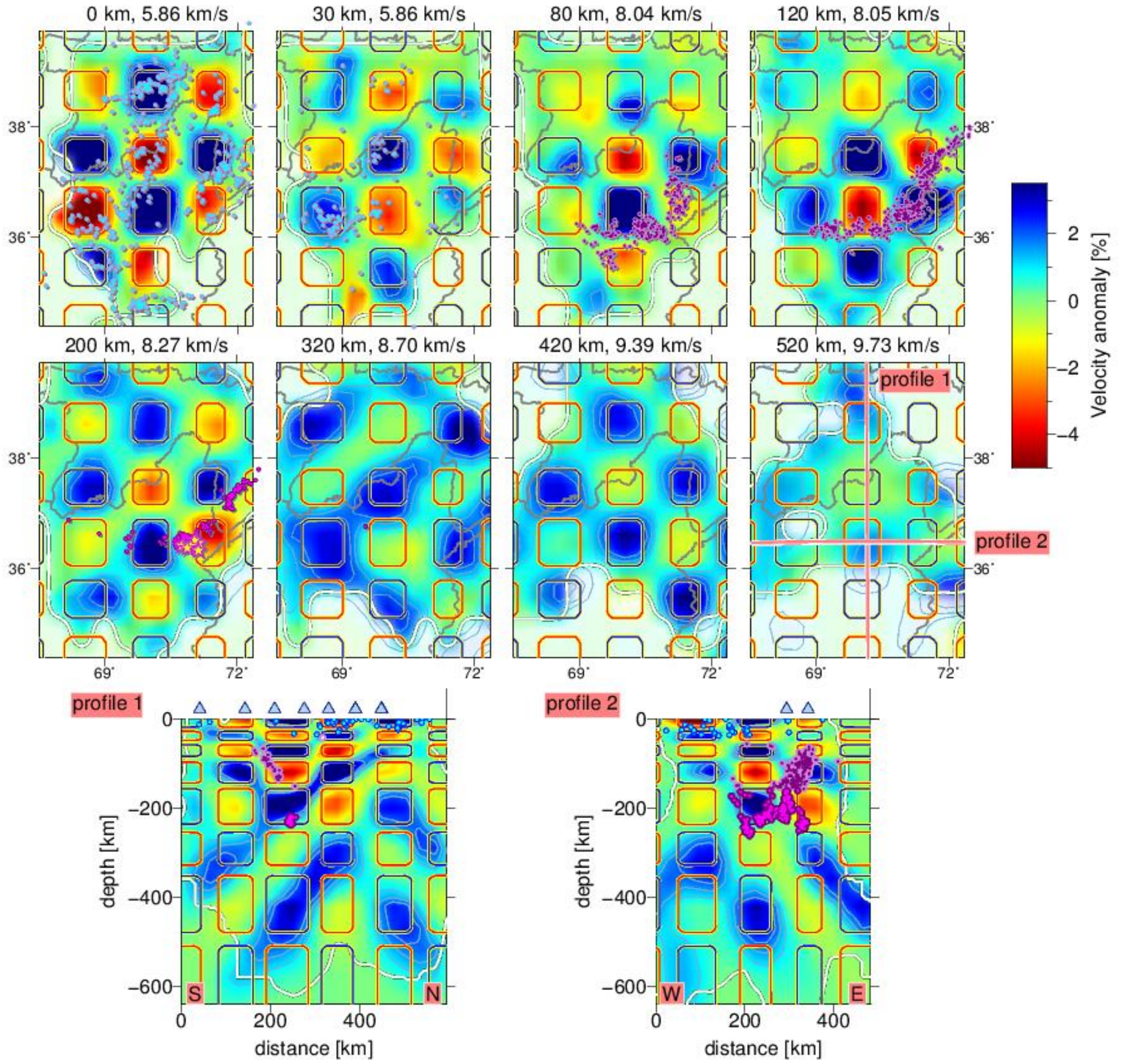

Supplementary Fig. 3 Checkerboard test. Synthetic test with a varying pattern of positive and negative velocity anomalies (±5%). Depth and reference velocity for each depth map is given on top of each sub-figure. The profile locations of profiles 1 and 2 are highlighted in the 520 km depth map. Map view dimension of the input anomalies is ~80 km. The anomalies are separated by ~20 km neutral zones. The depth extent of the anomalies increases with depth from 30 km at crustal levels to up to 130 km in the mantle. In contrast to the synthetic tests in Figs. 3 & 4, no noise is added to the synthetic data and hypocentres are not disturbed. This is because the checkerboard test serves mainly as a proxy for ray coverage but does not resample realistic velocity anomalies. Results show a decrease of amplitude intensity at mantle depths. This is due to the relatively small nature of the implemented anomalies (80 km horizontal extent), which are resolved by teleseismic rays only at these depths. Synthetic tests with more realistic anomaly configurations (Figs. 3 and 4) showed a better amplitude recovery. White outline, blue/pink/purple circles and triangles represent resolution limit, local earthquakes and seismic stations as in Figure 2. Political boundaries are plotted in grey. The contours of the input synthetic model are highlighted in blue (positive anomaly) and red (negative anomaly).

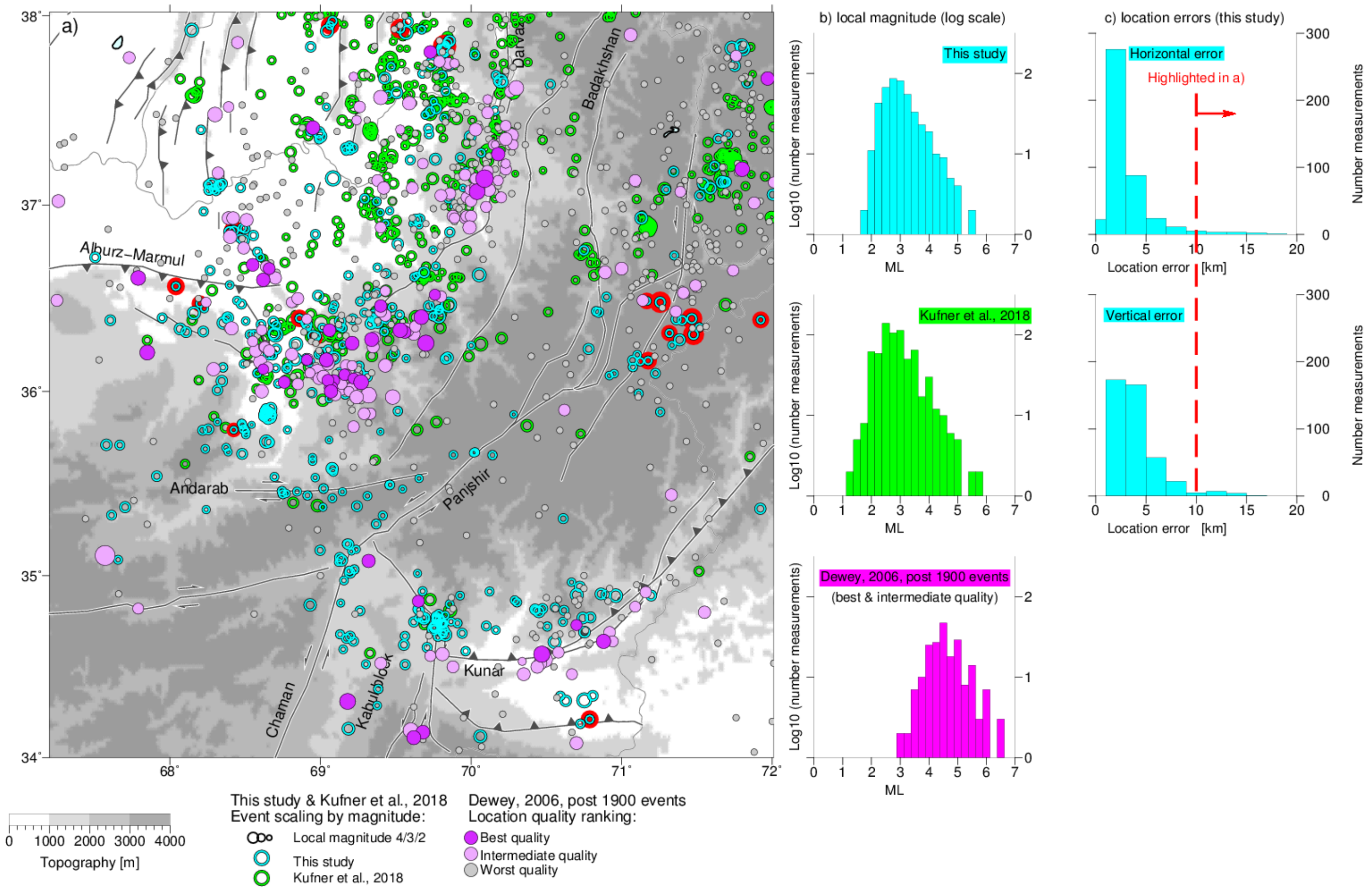

Supplementary Fig. 4 Comparison of crustal event catalogues. a) Comparison of our crustal event catalogue to ref. [6], a USGS compilation of published historic earthquake sources, and to ref. [7]. The ref. [7] catalogue was derived from a temporary seismic network located mainly in Tajikistan. The USGS compilation includes earthquakes of magnitude 5.5 and larger during 1900-63, and events with smaller magnitude since 1964. Dependent on quality, the USGS compilation groups events in three quality classes. For the best quality class, depth and horizontal location uncertainties are independent and less than 10 km. The worst quality contains events, which hypocentres have uncertain or unknown accuracy. The intermediate quality class includes all other events. Comparison of the herein presented event catalogue to the USGS compilation with longer observation duration suggests that the crustal seismicity pattern depends little on the observation period. Events highlighted in red are earthquakes derived in this study with location uncertainties larger than 10 km. b) Magnitude histograms for the three different event catalogues compared in a). The herein presented event catalogue contains more earthquakes at smaller magnitude compared to the USGS compilation, which highlights the advantage of a local seismic network in detecting such events. c) Location uncertainty histograms of the event catalogue derived herein, separated in vertical and horizontal errors.

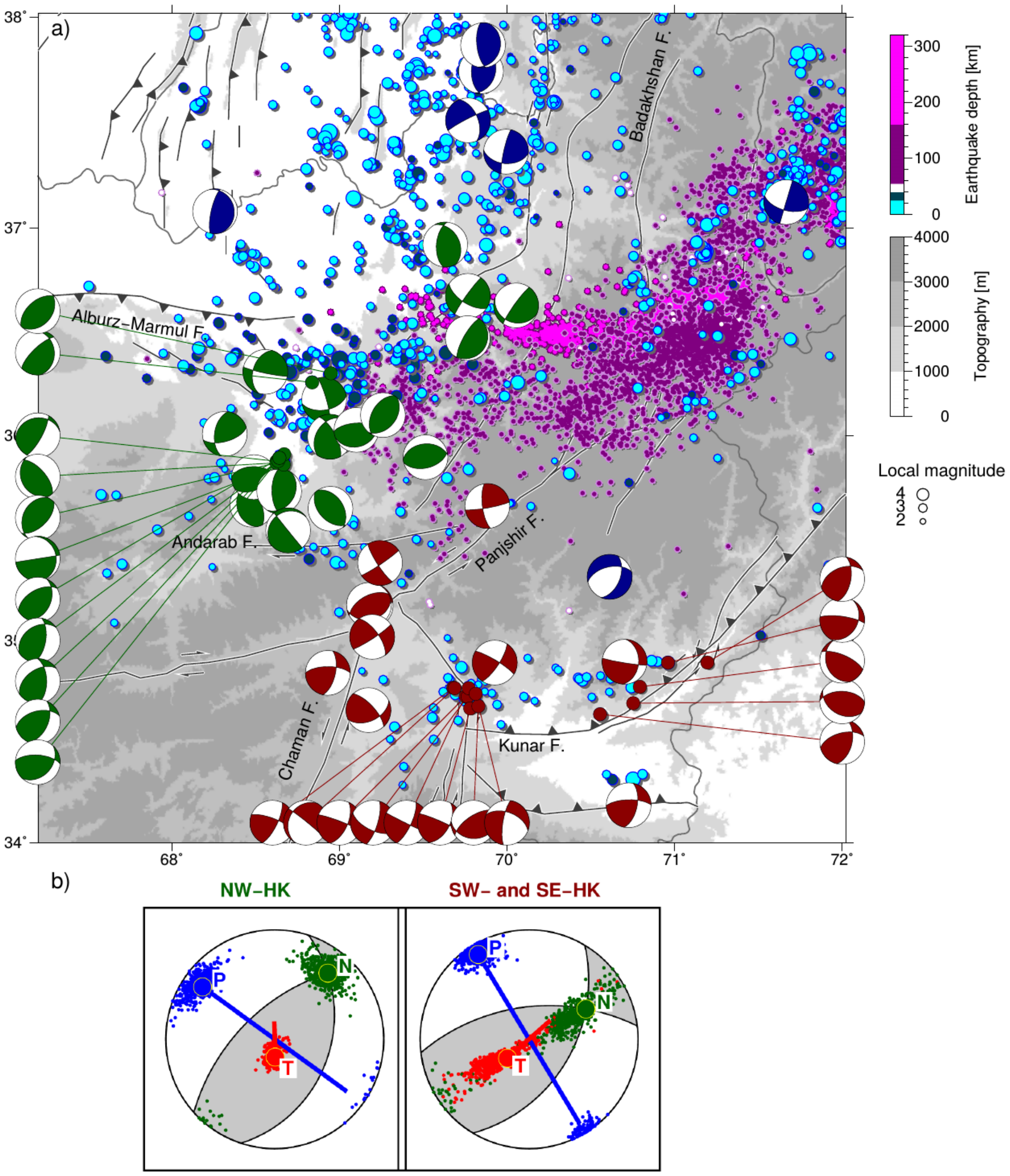

Supplementary Fig. 5 Stress inversion in sub-regions. a) Crustal focal mechanisms (0-40 km) derived from first motion polarities. Mechanisms used for stress inversion are highlighted in dark green (NW-HK in b) and dark red (SW- and SE-HK in b). All other plotted features as in Fig. 5b. b) Results of fault plane data inverted for stress tensors in the two sub-regions indicated in Supplementary Fig. 5a, plotted as beach-ball representations with highlighted compression-axes (P) and extension-axes (T). Small colour-coded circles show the spread of P-axes, T-axes and Null-axes (N) derived from bootstrap tests. The spread of these solutions is a measure for the robustness of the solution.

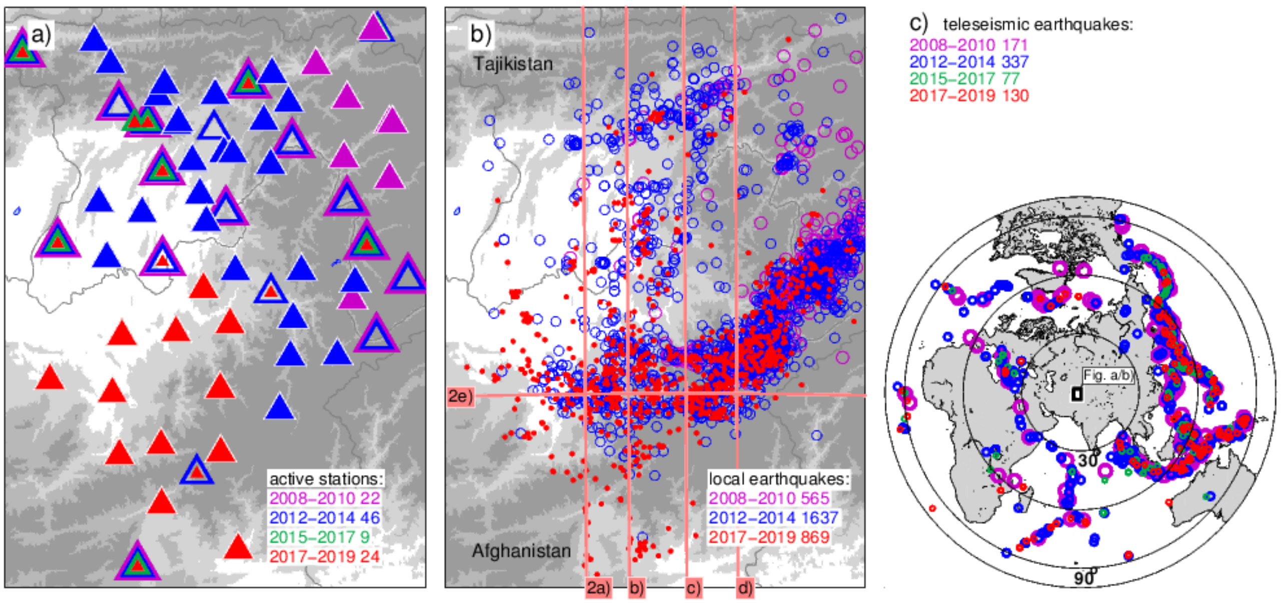

Supplementary Fig. 6 Input data for tomography separated by network deployment time. a) Seismic stations used for the inversion (including campaign and permanent stations), colour-coded by deployment phase. Inset lists the years during which the stations were active and the total number of stations during these periods. The font colours correspond to the symbol colours. b) Local earthquakes registered by the different networks shown in a). Cross-section locations of Figs. 2a-e are highlighted in light-red. c) Teleseismic earthquakes used for inversion, colour-coded by deployment phases – centred on the study region. The size of symbols plotted in a-c) does not visualize any scaling but serves only to discriminate nearby symbols.

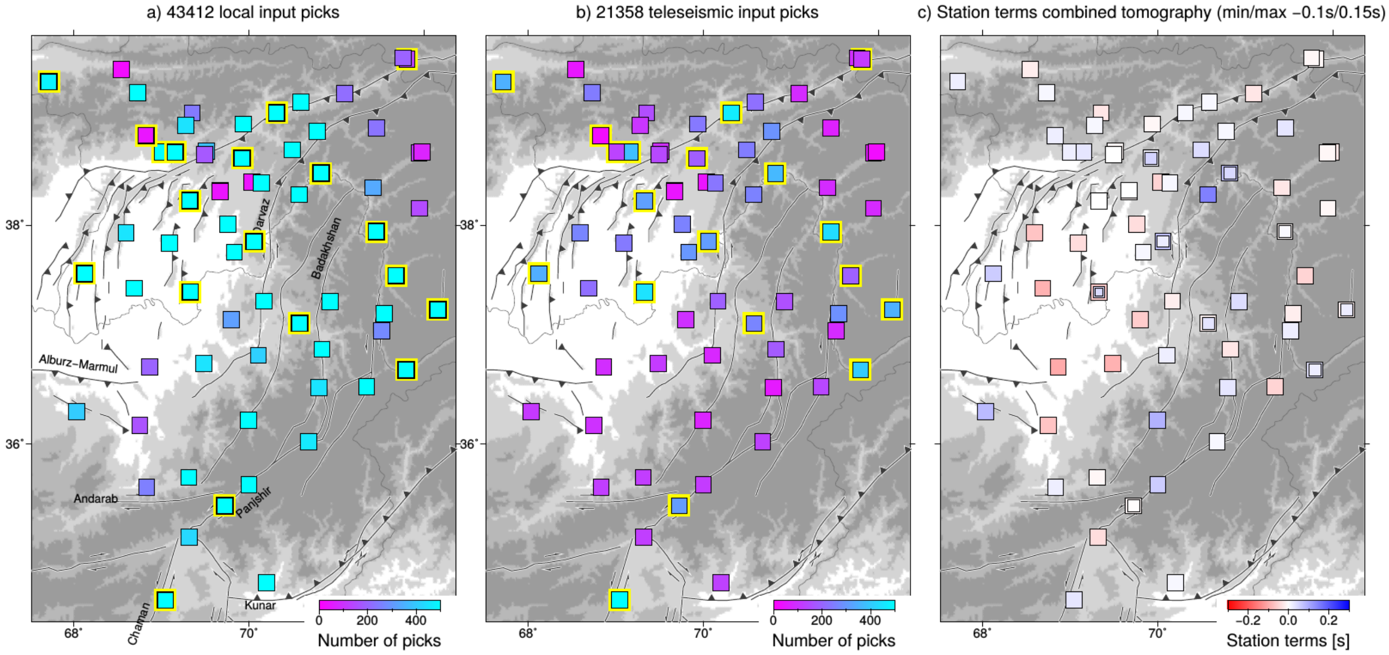

Supplementary Fig. 7 Input picks for inversion and output station terms. a/b) Stations are colour-coded by the number of local (a) and teleseismic (b) picks, respectively. Yellow boxes highlight stations active during multiple deployment periods. c) Station terms derived from the joint inversion. If a campaign station was re-deployed in different networks, the individual station terms are plotted on top of each other with squares of decreasing size.

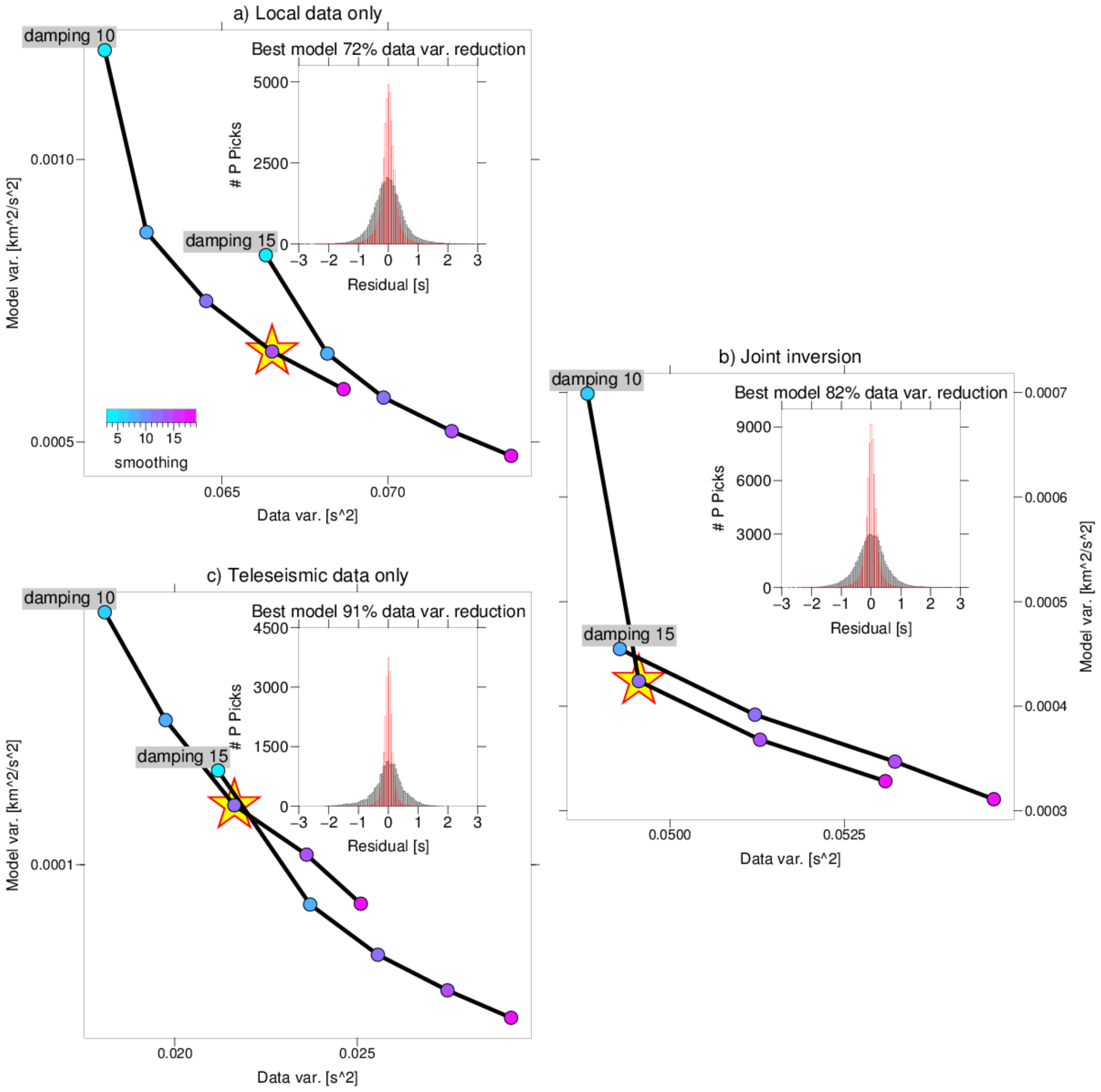

Supplementary Fig. 8 Trade-off curves between data variance and model variance dependent on different smoothing and damping values used in the inversion. Data variance is calculated from the travel time residuals after the final inversion step, model variance is the variance of the final velocity model in domains with hit-quality larger than 0.35. Each circle represents results of an individual model inversion with the colour-coding representing the smoothing value used. Note that local earthquake input used for the derivation of the L-curves varies slightly from the models shown in Figure 2, but output models are almost identical. The final set of parameters (highlighted as yellow star) was chosen as the best compromise between model roughness and data variance reduction. Insets show P-wave residuals prior to (grey) and after the final iteration (red) of the models shown in Figs. 2 and 3a. a) Model based on local data only. b) Combined teleseismic and local (joint) inversion. c) Model based on teleseismic data only.

Supplementary Tab. 1 Detailed explanation of local input data subsets

Network name<br><br>(FDSN network code)	Stations used for tomography	common stations with other networks	Selection criteria (local data)
TIPAGE (7B, 2008-2010)	22	14	Declustered catalogue used by ref. [1] for local earthquake tomography: (a) only events in the Hindu Kush and western Pamir are included; (b) for these events, the minimum number of picks for an earthquake to be included in the inversion is restricted dependent on the event depth:<br><br>- min. 10 picks required for crustal events<br><br>- min. 14 picks required for intermediate-depth events<br><br>(c) The catalogue was further declustered in the ~160 km - 220 km deep cluster of intermediate-depth seismicity. Declustering was done by allowing a maximum of two events (those with the largest number of picks) in 10 km cubes.
TIPTIMON Tajikistan (5C, 2012-2014), TIPTIMON Afghanistan<br><br>(6C, 2013-2014)	46	17	Declustered catalogue used by ref. [2] for local earthquake tomography. The minimum number of picks for an earthquake to be included in the inversion is restricted dependent on the epicentral position and event depth:<br><br>- min. 8 picks required for crustal events<br><br>- min. 20 picks required for intermediate-depth events in centre of network<br><br>- min. 14 picks required for intermediate-depth events at extremities of network<br><br>All events registered at stations in Afghanistan are kept. As for the TIPAGE catalogue, additional declustering between ~160 km and 220 km depth was implemented.
2015-2016	9	9	Only telseismic data recorded at permanent stations are used.
Afghanistan TIPTIMON II<br><br>(4C, 2017-2019)	24	12	Data first published here. An event catalogue was automatically derived following the location chain of ref. [8], then selection criteria were applied to obtain a high quality subset:<br><br>- at least 2 S picks and 5 P Picks with quality class better than 3*<br><br>- no rms greater than 1.5 s using the 1D velocity model of ref. [2]<br><br>- no P-picks with quality class 3*<br><br>As for the TIPAGE and TIPTIMON catalogues, additional declustering between ~160 km and 220 km depth was implemented.<br><br>After applying these selection criteria, the entire dataset was visually re-inspected in SeisComp3 and further picks were added. This step was introduced to increase the number of picks at stations in Afghanistan.

* see ref. [8] for a description of pick quality classes.

Supplementary References

1. Sippl, C. *et al.* Deep burial of Asian continental crust beneath the Pamir imaged with local earthquake tomography. *Earth Planet. Sci. Lett.* (2013) doi:10.1016/j.epsl.2013.10.013.
2. Kufner, S.-K. *et al.* Zooming into the Hindu Kush slab break-off: A rare glimpse on the terminal stage of subduction. *Earth Planet. Sci. Lett.* **461**, (2017).
3. Evans, J. R., Eberhart-Phillips, D. & Thurber, C. H. User's manual for SIMULPS12 for imaging Vp and Vp/Vs; a derivative of the" Thurber" tomographic inversion SIMUL3 for local earthquakes and explosions. *US Geol. Surv.* **94**–**431**, (1994).
4. Kufner, S.-K. *et al.* Deep India meets deep Asia: Lithospheric indentation, delamination and break-off under Pamir and Hindu Kush (Central Asia). *Earth Planet. Sci. Lett.* (2016) doi:10.1016/j.epsl.2015.11.046.
5. Koulakov, I. LOTOS code for local earthquake tomographic inversion: Benchmarks for testing tomographic algorithms. *Bull. Seismol. Soc. Am.* **99**, 194–214 (2009).
6. Dewey, J. Seismicity of Afghanistan and vicinity. *(Editor). US Dep. Inter. US Geol. Surv.* (2006).
7. Kufner, S.-K. *et al.* Seismotectonics of the Tajik Basin and Surrounding Mountain Ranges. *Tectonics* (2018) doi:10.1029/2017TC004812.
8. Sippl, C. *et al.* Geometry of the Pamir-Hindu Kush intermediate-depth earthquake zone from local seismic data. *J. Geophys. Res. Solid Earth* **118**, 1438–1457 (2013).
